# Breaching the Blood-Brain Barrier as a Gate to Psychiatric Disorder

**DOI:** 10.1155/2009/278531

**Published:** 2009-08-27

**Authors:** Hadar Shalev, Yonatan Serlin, Alon Friedman

**Affiliations:** ^1^Department of Psychiatry, Soroka University Medical Center; Zlotowski Center for Neuroscience, Ben-Gurion University of the Negev, Beer-Sheva 84105, Israel; ^2^Department of Physiology and Neurosurgery, Soroka University Medical Center; Zlotowski Center for Neuroscience, Ben-Gurion University of the Negev, Beer-Sheva 84105, Israel

## Abstract

The mechanisms underlying the development and progression of psychiatric illnesses are only partially known. Clinical data suggest blood-brain barrier (BBB) breakdown and inflammation are involved in some patients groups. Here we put forward the “BBB hypothesis” and abnormal blood-brain communication as key mechanisms leading to neuronal dysfunction underlying disturbed cognition, mood, and behavior. Based on accumulating clinical data and animal experiments, we propose that events within the “neurovascular unit” are initiated by a focal BBB breakdown, and are associated with dysfunction of brain astrocytes, a local inflammatory response, pathological synaptic plasticity, and increased network connectivity. Our hypothesis should be validated in animal models of psychiatric diseases and BBB breakdown. Recently developed imaging approaches open the opportunity to challenge our hypothesis in patients. We propose that molecular mechanisms controlling BBB permeability, astrocytic functions, and inflammation may become novel targets for the prevention and treatment of psychiatric disorders.

## 1. Structure and Function of the Blood-Brain Barrier

More than a century ago, Ehrlich [1] demonstrated the lack of permeability of intracerebral vessels to albumin-binding dyes, which suggested the presence of a barrier to proteins in the brain. Several years later, Lewandowsky [2] suggested that the absence of central nervous system (CNS) pharmacological actions of intravenously administered bile acids or ferrocyanide was due to the blood-brain barrier (BBB), a mechanical membrane that separates blood from brain. 

Three barrier layers regulate molecular exchange at the interfaces between the blood and the neural tissue or its fluid spaces: the BBB formed by the cerebrovascular endothelial cells between blood and brain interstitial fluid, the choroid plexus epithelium between blood and ventricular cerebrospinal fluid (CSF), and the arachnoid epithelium between blood and subarachnoid CSF. Since individual neurons are extremely close to the brain capillaries, rarely at a distance greater than 20 *μ*m, of the various CNS barriers, the BBB exerts the greatest control over the immediate microenvironment of brain cells [3]. The BBB is present in the capillaries throughout the brain (for reviews see [4–6]). The components composing the BBB include endothelial cells lining the lumen of brain capillaries, linked together by tight junctions that are composed of specific proteins (e.g., claudins, occludins, ZO-1, ZO-2, ZO-3, and cingulin—see [7]), pinocytic vesicles, and specific transport mechanisms. Surrounding the endothelial cells are the basement membrane, brain pericytes, and astroglial foot processes—creating a third continuous layer that separates these blood vessels from the brain tissue. All of these components enable the formation of a barrier that hinders the entry of most molecules into the brain, and that is actively involved in exporting such molecules out of the brain in case of penetration. With the exception of very small lipophilic molecules, hydrophilic molecules enter the brain via active transport, and specific transporters exist for required nutrients such as glucose and certain amino acids.

## 2. Blood-Brain Barrier Breakdown in Diseases

In numerous pathologies of the brain, and also in other vascular, inflammatory, and infectious diseases, the function of the BBB is often compromised [3]. For example, the capillaries of many glial tumors are leaky compared to those of normal brain tissue, either as a result of a lack of inductive factors, or owing to the release of permeability factors such as vascular endothelial growth factor (VEGF). Moreover, the tight junction protein claudin 3 is downregulated in some brain tumors [8]. In addition, evidence for the loss of agrin from the abluminal surface of the endothelial cells adjacent to astrocytic endfeet [9] may contribute to BBB damage in Alzheimer's disease [10] and to the redistribution of astrocytic Aquaporin 4 (AQP4) in glioblastomas [11]. AQP4 and inward rectifying potassium channels (K_IR_4.1) are often downregulated in BBB breakdown [12] and may contribute to the lack of homeostasis for water and potassium molecules (described hereafter). Some neuropathologies, such as multiple sclerosis and epilepsy, may involve an early phase of BBB disturbance that precedes the disorganization and damage of the neuronal network, suggesting that vascular damage and increased BBB permeability can lead to secondary neuronal disorders [13–15]. In epilepsy, the normal pattern of brain ATP-binding cassette (ABC) transporters expression may also change, with upregulation of P-glycoprotein (Pgp) on astrocytes and the brain endothelium [16]; this may lead to the efflux of antiepileptic drugs from the brain into the vascular compartment and contribute to a pharmacoresistent disease. Kortekaas et al. [17] showed an elevated uptake of the Pgp substrate [11C]verapamil using positron emission tomography (PET) in the midbrain of patients with Parkinson's disease, which is consistent with disturbed Pgp function in the BBB. In animal models of Alzheimer's disease, amyloid-*β* (A*β*) accumulation is often first seen in close proximity to blood vessels, with toxicity on endothelium and astrocytes observed before significant neuronal loss [18]. The ability of agents released during inflammation to increase the permeability of the brain endothelium has been described. Activation of endothelial bradykinin B2 receptors causes an increase in intracellular Ca^2+^ concentrations and tight junctions opening and may underlie BBB opening in conditions in which intravascular coagulation occurs. In addition, bradykinin can activate NF-*κ*B pathway in astrocytes, leading to the release of interleukin-6 (IL-6), which can amplify the effect by acting back on the endothelium [19]. Tumor necrosis factor-*α* (TNF*α*) increases BBB permeability by direct action on the endothelium [20] and indirectly via endothelin 1 production and IL-1*β* release from astrocytes [21]. During injury, several substances are released from central and peripheral neurons, connective tissue, cells, and blood cells. Many of these substances, such as matrix metaloproteases, substance P, calcitonin gene-related peptide (CGRP), serotonin, histamine, and ATP, can affect BBB permeability. For example, the release of IL-1*β* leads to a decreased concentration or altered localization of the tight junction protein occludin, and increases BBB permeability. TNF*α*, histamine and interferon-*γ* released in inflammatory pain can also cause changes in brain endothelial permeability [22]. Massive neuronal activation, as observed under spreading depolarization, may induce upregulation and release of metaloproteases causing BBB breakdown [23].

## 3. From BBB Breakdown to Psychopathology

We hypothesize that a primary vascular pathology, leading to BBB breakdown, will result in the leakage of serum-derived vascular components into the brain tissue and may cause brain dysfunction which, under some conditions (extent, duration, and/or location), will result in disturbed thinking processes, mood, and behavior—such as those characterizing psychiatric patients. Here we will present clinical evidence for BBB pathology in psychiatric patients and hypothesize potential mechanisms linking altered vascular permeability to psychopathology.

### 3.1. Clinical Evidence

Clinical evidence for BBB breakdown in patients with psychopathology is insufficient. However, accumulating data strongly suggest the existence of BBB pathology in a subset of patients with major psychiatric illnesses, including depression and schizophrenia. The main difficulties are, on the one hand, the lack of a routine, quantitative, and reproducible comprehensive method for measuring BBB permeability in human, and on the other hand, the lack of reliable and widely accepted animal models for human psychiatric illnesses.

Qualitative evaluation of BBB disruption in patients is most frequently conducted using common brain imaging modalities, such as magnetic resonance imaging (MRI), computerized tomography (CT), and single photon emission CT (SPECT), following the peripheral administration of nonpermeable contrast agents [24]. Extravascular accumulation of the contrast agent indicates BBB breakdown. Although CT and MRI are the best available methods for studying anatomical brain lesions and are most often used as diagnostic tools (mainly to exclude other pathologies) in psychiatric patients, the currently used imaging protocols are relatively insensitive in detecting small changes in contrast agent accumulation (e.g., as compared with SPECT [25]). Bilateral contrast (gadolinium) enhancement of the temporal lobes in the region of the amygdala and hippocampus has been reported in a case report of a patient with progressive insomnia, short-term memory loss, depression, and cognitive impairment due to paraneoplastic syndrome [26]. In order to increase method sensitivity and allow quantification of BBB permeability, several methods has recently been developed using dynamic contrast enhanced imaging (e.g., [27, 28], and see also the review by Zaharchuk [29]). These methods have not yet become available for routine clinical examinations, and published studies relate a relatively small number of patients with brain tumors or posttraumatic epilepsy. To this end, there are no published studies in psychiatric patients.

Quantitative evaluation of BBB functioning in the clinical setting can also be obtained using analysis of the cerebrospinal fluid (CSF) for serum proteins (e.g., albumin) or plasma analysis of brain constituents (e.g., S100B, [30]). However, neither of these measures is routinely assessed in patients, and only incomplete data from small clinical studies are available. BBB dysfunction has been indicated in a subgroup of schizophrenic patients by measuring increased albumin and IgG CSF levels [31, 32]. Muller and Ackenheil [31] further showed an association between CSF-serum albumin ratio and the patients' negative symptoms. CSF-serum albumin ratio was also found to be higher in elderly depressed women [33] and in patients suffering from dementias compared to age-matched controls [34]. Interestingly, nondemented women who developed dementia during the research follow-up had an initial higher CSF-serum albumin ratio compared to those who did not develop the disease, suggesting BBB dysfunction may precede the clinical symptoms and be related to the pathogenesis of the disease. These clinical studies are supported by morphological studies demonstrating abnormalities in cerebral capillaries that bear BBB functions. These include degeneration and focal necrotic changes of the endothelium, vascular basement membrane alterations accompanied by accumulation of collagen fibrils, decreased mitochondrial content, as well as increased pinocytotic vesicles and loss of tight junctions [35, 36].

S100B is primarily a brain-specific, astrocytic calcium binding protein involved in intracellular signal transduction. Information about the functional implication of S100B secretion by astrocytes is scant, but accumulating evidence suggests it exerts trophic or toxic effects depending on its concentration [37]. Accumulating data from clinical studies convincingly demonstrate increased S100B levels in the serum of patients suffering from acute or chronic schizophrenia as well as depression [38, 39]. The observed increase in plasma levels of S100B may reflect increased BBB permeability [30]. However, increased plasma levels of S100B may also be due to increased production and/or active secretion from glia cells, or to their destruction [40, 41].

### 3.2. BBB-Induces Astrocytic Transformation and Dysfunction

Astrocytic end-feet surrounding capillaries in the central nervous system have a role in regulating tight junction expression [42], and are considered an integral part of the BBB. However, BBB breakdown results in a rapid (within hours) transformation of the normally occurring “resting astrocytes” into “active” ones [13]. While the signaling mechanisms underlying astrocytic transformation in the injured brain are not known, recent experimental evidence indicates that extravasation of the most abundant serum protein, albumin, into the neuropil may serve as the key factor for astrocytic transformation via the transforming growth factor beta (TGF*β*) signaling pathway. Transformed astrocytes are observed in ischemic, inflammatory, and traumatic brain injuries and are often the only pathological change observed in the epileptic brain. Transformed astrocytes are characterized by a robust change in gene expression that includes the upregulation of GFAP and S100B, downregulation of glutamate transporters and glutamine synthase, as well as the inward rectifying potassium channel, K_IR_4.1, AQP4, and gap junctions' proteins (see [43] and unpublished data). These robust changes in gene expression predict a dysregulation in homeostasis of the extracellular environment, specifically increased concentrations of potassium and glutamate—which are increased with neuronal activity and strongly affect their excitability [12, 43]. In addition, experimental evidence indicates that transformed astrocytes upregulate the expression of cytokines and are part of the local inflammatory brain response (described hereafter).

Is there evidence for the transformation of astrocytes in the brains of psychiatric patients? Brain injuries that often involve BBB breakdown and astrocytic response increase the risk for significant neuropsychiatric sequelae, including personality changes, depression, anxiety, dementia, and perhaps psychosis [44, 45]. The hypothalamic-pituitary-adrenal (HPA) axis is activated by stress, and its activity is seen under acute and chronic stress conditions, including mental and physiological distress. Stress activates the HPA axis through the release of corticotropin-releasing hormone (CRH), leading to secretion of catecholamines and glucocorticoids. CRH has proinflammatory effects that are mediated through mast cells, which regulate BBB permeability. Indeed, stress has been shown to induce BBB breakdown [46, 47], which can be blocked by pretreatment with the CRH receptor antagonist antalarmin [48]. Interestingly, antalarmin has been also shown to block stress-induced behavioral changes.

Recently, direct evidence support the presence of an astrocytic response in the brains of psychiatric patients even with no obvious preceding injury. Most studies based the “astrocytic hypothesis” of schizophrenia and depression on increased serum or CSF levels of the astrocytic S100B protein. In summarizing the results of clinical studies, reports consistently show increased S100B levels in patients suffering from schizophrenia in both the acute and chronic psychotic stages of disease. Furthermore, this increase remains in those patients who develop a residual state with relevant negative symptoms, whereas S100B normalizes in recovering patients. Neuroleptic medication most likely cannot be regarded as a confounding factor, as these findings have been reported in nonmedicated patients as well (for review, see [41]). While these studies did not differentiate between increased S100B production and increased BBB permeability for S100B (when measured in serum), in a recent histological study Steiner et al. [49] analyzed the cell-density of S100B-immunopositive glia in different brain regions (including the anterior cingulate, dorsolateral prefrontal (DLPF), orbitofrontal, superior temporal cortex, and hippocampus) of 18 patients with schizophrenia (16 matched controls). They reported that cortical brain regions contained significantly more S100B-immunopositive glia in the schizophrenia group compared to controls. This effect was pronounced in the paranoid schizophrenia subgroup and particularly in the DLPF brain area. Glial pathology has also recently been demonstrated in mood disorders. In a combined clinical study and a meta-analysis of published studies on S100B involving 193 patients suffering from mood disorders and 132 healthy controls [39], the authors concluded that serum levels of S100B are consistently elevated during acute major depressive or manic episodes. Additionally, it is demonstrated that serum S100B decreases reliably during antidepressive treatment together with clinical improvement.

### 3.3. BBB Breakdown, Astrocytic Transformation, and Brain Inflammation

There is strong clinical evidence in subgroups of patients with depression that symptom intensity and the course of disease are associated with elevated plasma levels of pro-inflammatory mediators, including IL-1, IL-2, IL-6, TNF, and C-reactive protein (for review, see [50]). Multiple studies have shown that there is a decrease in Th1 mediator levels with antidepressant treatment, indicating that one potential mechanism of action of antidepressant treatments is decreased inflammation. In a rare prospective study [51], an increased inflammatory state at baseline (elevated levels of C-reactive protein and increased capacity of leukocytes to produce IL-1) predicted later onset of depression in elderly individuals without a prior history of depression, suggesting that excess inflammation precedes depression. It is not as yet entirely clear how inflammatory cytokines affect mood and high cognitive functions.

Another clue for immune-to-brain communication associated with psychpathology may be gleaned from patients with chronic neuropathic pain. Activation of phagocytic immune cells (e.g., macrophages) around an otherwise healthy peripheral nerve leads to the release of proinflammatory cytokines (TNF, IL1, and IL6), as well as other proinflammatory substances [52]. The inflammatory response may be a link to the high prevalence of depression among these patients.

Thus, we hypothesize that primary BBB breakdown will enhance brain inflammation through two main mechanisms. (1) In the event of a peripheral inflammatory response, BBB breakdown will increase the transport of peripheral cytokines into the brain, thus inducing the activation of astrocytes, microglia, and recruitment of immune cells. (2) Prolonged and pronounced BBB breakdown will directly induce a local inflammatory response by activating astrocytes (as explained above) that will secrete cytokines locally.

Increased cytokine levels may also be involved in depression via their direct effect on serotonin levels [53]. Inflammatory mediators may also alter network properties and neuronal excitability by changing glutamate levels and affecting synaptic plasticity: inflammatory mediators can, through activation of the kynurenine pathway, increase kynurenic acid—an NMDA receptor antagonist, and quinolinic acid—an NMDA receptor agonist. Microglia are the only cells in the central nervous system that express the complete enzymatic pathway required for the synthesis of quinolinic acid [54]. Therefore, inflammatory mediators acting on microglia will increase the ratio of quinolinic acid to kynurenic acid, leading to increased activation of NMDA receptors. Recent studies further suggest that increased glutamate levels may also activate astrocytes and microglia leading to release of inflammatory mediators, causing a vicious cycle.

### 3.4. From BBB Breakdown via Astrocytic Transformation and Inflammatory Response to Brain Dysfunction

Our hypothesis directly links BBB breakdown, the consequent transformation of astrocytes and microglia, and the resultant local inflammation to network dysfunction, which may underlie psychiatric illnesses. As mentioned earlier, the dysfunction of astrocytes and the inflammatory response are expected to cause a reduction in the buffering of extracellular potassium and glutamate. Together with increased quinolinic acid, these will act synergistically to depolarize neuronal membranes, thus further allowing the activation of the voltage-dependent NMDA receptors. While in extreme cases the excess of NMDA receptors activation may lead to neuronal toxicity, it is important to point out that neurotoxicity in psychiatric disorders has not been unequivocally demonstrated. We propose that under milder conditions, enhancing NMDA receptors activation results in abnormal synaptic plasticity—which refers to enhanced synaptic strength and (or) loss of pathway specificity. Such reduced specificity is expected to result in the activation of larger neuronal networks in response to stimuli, Manifested as disturbed thinking processes and extreme mood-related behavioral responses, depending on the involved network. Under even more extreme conditions, the activated network will be larger, leading to epileptic activity [13] and delayed neuronal toxicity [14].

Interestingly, a recent study demonstrated that in the KA rat model for schizophrenia, there is suppression of homosynaptic long-term plasticity with disturbed heterosynaptic depression within the hippocampus [55]. Increased connectivity between neighboring neurons has been also found in the valopric acid model of autism in rats [56]. The authors suggested that this local hyperconnectivity may render cortical modules more sensitive to stimulation and, once activated, make them more autonomous, isolated, and more difficult to command.

## 4. Summary and Future Perspectives

**Figure 1:**
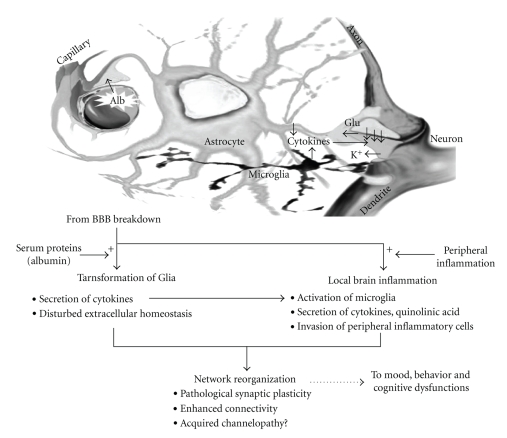
*The BBB Hypothesis of Psychiatric Disorders*. Interactions within the neurovascular unit in the presence of abnormal blood-brain communication. BBB breakdown results in the diffusion of serum proteins (e.g., albumin, see text) into the brain, activation of signaling pathways, and inducing the transformation of astrocytes. This “reactive” glial reaction is associated with impaired extracellular homeostasis (e.g., reduced buffering of extracellular potassium and glutamate) and a local inflammatory response (e.g., secretion of cytokines, activation of microglia) and is enhanced in the presence of an inflammation in the periphery. Together, neuronal network dysfunction develops due to pathological synaptic plasticity.

Figure 1 summarizes the sequence of events occurring within the “neurovascular unit,” which according to the proposed hypothesis leads to network hyperconnectivity and psychiatric illness. According to our hypothesis, early injury to the BBB, due to vascular or brain pathology, will disturb “blood-brain communication” and initiate a sequence of events that will finally result in abnormal plasticity within the neuronal network, namely, hyperconnectivity. Our hypothesis put together findings observed in psychiatric patients to form a broader pathophysiological picture. These findings include, for example, increased CSF albumin and serum S100B—as markers for BBB breakdown, increased brain astroglial markers within the brain—as markers for their activation, inflammatory markers—the result or cause of astroglial activation, and changes in glutamate levels, neuronal connectivity, and, in some cases, neurons loss. The hypothesis further explains the high rate of psychiatric illnesses following brain injury, acute stress reactions, and vascular pathologies (e.g., systemic lupus erythematosus) which may frequently be associated with BBB breakdown. It may also explain the relation between the presence of peripheral inflammatory process in some psychiatric patients as well as the frequent comorbidity with epilepsy—characterized by hyperexcitable and hypersynchronized network.

Our hypothesis raises many questions regarding the location (where?), extent (to which molecules?) and duration of BBB damage sufficient to induce the described disease process. We put forward testable predictions in animal models and human patients and raise the importance of time line, long-term, and, whenever possible, prospective studies, to challenge the proposed hypothesis. A better understanding of BBB damage and repair processes and serum-born signaling molecules is essential for the identification of new therapeutic targets. Our hypothesis calls for the use of recently developed approaches for measuring BBB breakdown in patients at risk for developing psychiatric illnesses (or during the early phases of the disease). The use of novel molecular markers for imaging brain inflammation may, in the future, facilitate better understanding of their role in different patient populations and at different stages of the disease. Future studies are also needed to uncover the interactions between BBB-mediated brain pathologies and specific transmitter systems which have been reported to play a role in specific psychopathologies. We further believe that our hypothesis—if found valid—will offer previously unforeseen targets for the prevention and treatment of psychiatric diseases. It may also explain and encourage the use of anti-inflammatory agents against psychosis, at some early stages of the disease [7–9]. Astrocytic dysfunction may also become a promising target at some point during the development of the disease.

In summary, the “BBB hypothesis” for impaired neuronal network connectivity is based on well-described interactions between components at the “neurovascular unit” and proposes a new concept pointing to disturbed blood-brain communication as a potentially important cause and future target for the treatment of psychiatric disorders.

## References

[1] Ehrlich P (1885). Das Sauerstoffbeduerfnis des Organismus: Eine Farbenanalytiche Studie. *Hirschwald*.

[2] Lewandowsky M (1900). Zur lehre der cerebrospinalflussigkeit. *Zeitschrift Klinische Medizin*.

[3] Abbott NJ, Ronnback L, Hansson E (2006). Astrocyte-endothelial interactions at the blood-brain barrier. *Nature Reviews Neuroscience*.

[4] Pardridge WM (1999). Blood-brain barrier biology and methodology. *Journal of Neurovirology*.

[5] Soreq H, Kaufer D, Friedman A, Glick C (2000). Blood-brain-barrier modulation and low-level exposure to xenobiotics.

[6] Zlokovic BV (2008). The blood-brain barrier in health and chronic neurodegenerative disorders. *Neuron*.

[7] Kniesel U, Wolburg H (2000). Tight junctions of the blood-brain barrier. *Cellular and Molecular Neurobiology*.

[8] Wolburg H, Wolburg-Buchholz K, Kraus J (2003). Localization of claudin-3 in tight junctions of the blood-brain barrier is selectively lost during experimental autoimmune encephalomyelitis and human glioblastoma multiforme. *Acta Neuropathologica*.

[9] Wolburg H, Lippoldt A (2002). Tight junctions of the blood-brain barrier: development, composition and regulation. *Vascular Pharmacology*.

[10] Berzin TM, Zipser BD, Rafii MS (2000). Agrin and microvascular damage in Alzheimer's disease. *Neurobiology of Aging*.

[11] Warth A, Mittelbronn M, Wolburg H (2005). Redistribution of the water channel protein aquaporin-4 and the K^+^ channel protein Kir4.1 differs in low- and high-grade human brain tumors. *Acta Neuropathologica*.

[12] Ivens S, Kaufer D, Flores LP (2007). TGF-*β* receptor-mediated albumin uptake into astrocytes is involved in neocortical epileptogenesis. *Brain*.

[13] Seiffert E, Dreier JP, Ivens S (2004). Lasting blood-brain barrier disruption induces epileptic focus in the rat somatosensory cortex. *Journal of Neuroscience*.

[14] Tomkins O, Friedman O, Ivens S (2007). Blood-brain barrier disruption results in delayed functional and structural alterations in the rat neocortex. *Neurobiology of Disease*.

[15] van Vliet EA, da Costa AS, Redeker S, van Schaik R, Aronica E, Gorter JA (2007). Blood-brain barrier leakage may lead to progression of temporal lobe epilepsy. *Brain*.

[16] Loscher W, Potschka H (2005). Drug resistance in brain diseases and the role of drug efflux transporters. *Nature Reviews Neuroscience*.

[17] Kortekaas R, Leenders KL, van Oostrom JCH (2005). Blood-brain barrier dysfunction in parkinsonian midbrain in vivo. *Annals of Neurology*.

[18] Iadecola C (2004). Neurovascular regulation in the normal brain and in Alzheimer's disease. *Nature Reviews Neuroscience*.

[19] Schwaninger M, Sallmann S, Petersen N (1999). Bradykinin induces interleukin-6 expression in astrocytes through activation of nuclear factor-*κ*B. *Journal of Neurochemistry*.

[20] Deli MA, Descamps L, Dehouck MP (1995). Exposure of tumor necrosis factor-*α* to luminal membrane of bovine brain capillary endothelial cells cocultured with astrocytes induces a delayed increase of permeability and cytoplasmic stress fiber formation of actin. *Journal of Neuroscience Research*.

[21] Didier N, Romero IA, Créminon C, Wijkhuisen A, Grassi J, Mabondzo A (2003). Secretion of interleukin-1*β* by astrocytes mediates endothelin-1 and tumour necrosis factor-*α* effects on human brain microvascular endothelial cell permeability. *Journal of Neurochemistry*.

[22] Huber JD, Egleton RD, Davis TP (2001). Molecular physiology and pathophysiology of tight junctions in the blood -brain barrier. *Trends in Neurosciences*.

[23] Gursoy-Ozdemir Y, Qiu J, Matsuoka N (2004). Cortical spreading depression activates and upregulates MMP-9. *The Journal of Clinical Investigation*.

[24] Avivi E, Tomkins O, Korn A, Pavlovsky L, Shelef I, Friedman A, Silman I, Fisher A, Anglister L, Michaelson D, Soreq H (2004). Blood-brain-barrier disruption in humans: a window to neurodegenerative diseases. *Cholinergic Mechanisms*.

[25] Volkow ND, Rosen B, Farde L (1997). Imaging the living human brain: magnetic resonance imaging and positron emission tomography. *Proceedings of the National Academy of Sciences of the United States of America*.

[26] Deodhare S, O'Connor P, Ghazarian D, Bilbao JM (1996). Paraneoplastic limbic encephalitis in Hodgkin's disease. *Canadian Journal of Neurological Sciences*.

[27] Tofts PS, Brix G, Buckley DL (1999). Estimating kinetic parameters from dynamic contrast-enhanced T(1)-weighted MRI of a diffusable tracer: standardized quantities and symbols. *Journal of Magnetic Resonance Imaging*.

[28] Tomkins O, Shelef I, Kaizerman I (2008). Blood-brain barrier disruption in post-traumatic epilepsy. *Journal of Neurology, Neurosurgery and Psychiatry*.

[29] Zaharchuk G (2007). Theoretical basis of hemodynamic MR imaging techniques to measure cerebral blood volume, cerebral blood flow, and permeability. *American Journal of Neuroradiology*.

[30] Marchi N, Rasmussen P, Kapural M (2003). Peripheral markers of brain damage and blood-brain barrier dysfunction. *Restorative Neurology and Neuroscience*.

[31] Muller N, Ackenheil M (1995). Immunoglobulin and albumin content of cerebrospinal fluid in schizophrenic patients: relationship to negative symptomatology. *Schizophrenia Research*.

[32] Schwarz MJ, Ackenheil M, Riedel M, Muller N (1998). Blood-cerebrospinal fluid barrier impairment as indicator for an immune process in schizophrenia. *Neuroscience Letters*.

[33] Gudmundsson P, Skoog I, Waern M (2007). The relationship between cerebrospinal fluid biomarkers and depression in elderly women. *American Journal of Geriatric Psychiatry*.

[34] Skoog I, Wallin A, Fredman P (1998). A population study on blood-brain barrier function in 85-year-olds: relation to Alzheimer's disease and vascular dementia. *Neurology*.

[35] Harik SI, Kalaria RN (1991). Blood-brain barrier abnormalities in Alzheimer's disease. *Annals of the New York Academy of Sciences*.

[36] Kalaria RN (1992). The blood-brain barrier and cerebral microcirculation in Alzheimer disease. *Cerebrovascular and Brain Metabolism Reviews*.

[37] Sen J, Belli A (2007). S100B in neuropathologic states: the CRP of the brain?. *Journal of Neuroscience Research*.

[38] Rothermundt M, Ponath G, Glaser T, Hetzel G, Arolt V (2004). S100B serum levels and long-term improvement of negative symptoms in patients with schizophrenia. *Neuropsychopharmacology*.

[39] Schroeter ML, Abdul-Khaliq H, Krebs M, Diefenbacher A, Blasig IE (2008). Serum markers support disease-specific glial pathology in major depression. *Journal of Affective Disorders*.

[40] Rothermundt M, Falkai P, Ponath G (2004). Glial cell dysfunction in schizophrenia indicated by increased S100B in the CSF. *Molecular Psychiatry*.

[41] Rothermundt M, Ponath G, Arolt V (2004). S100B in schizophrenic psychosis. *International Review of Neurobiology*.

[42] Haseloff RF, Blasig IE, Bauer HC, Bauer H (2005). In search of the astrocytic factor(s) modulating blood-brain barrier functions in brain capillary endothelial cells in vitro. *Cellular and Molecular Neurobiology*.

[43] Friedman A, Kaufer D, Heinemann U (2009). Blood-brain barrier breakdown-inducing astrocytic transformation: novel targets for the prevention of epilepsy. *Epilepsy Research*.

[44] Fleminger S (2008). Long-term psychiatric disorders after traumatic brain injury. *European Journal of Anaesthesiology*.

[45] Guerreiro DF, Navarro R, Silva M, Carvalho M, Gois C (2009). Psychosis secondary to traumatic brain injury. *Brain Injury*.

[46] Sharma HS, Dey PK (1981). Impairment of blood-brain barrier (BBB) in rat by immobilization stress: role of serotonin (5-HT). *Indian Journal of Physiology and Pharmacology*.

[47] Friedman A, Kaufer D, Shemer J, Hendler I, Soreq H, Tur-Kaspa I (1996). Pyridostigmine brain penetration under stress enhances neuronal excitability and induces early immediate transcriptional response. *Nature Medicine*.

[48] Esposito P, Chandler N, Kandere K (2002). Corticotropin-releasing hormone and brain mast cells regulate blood-brain-barrier permeability induced by acute stress. *Journal of Pharmacology and Experimental Therapeutics*.

[49] Steiner J, Bernstein HG, Bielau H (2008). S100B-immunopositive glia is elevated in paranoid as compared to residual schizophrenia: a morphometric study. *Journal of Psychiatric Research*.

[50] Dantzer R, O'Connor JC, Freund GG, Johnson RW, Kelley KW (2008). From inflammation to sickness and depression: when the immune system subjugates the brain. *Nature Reviews Neuroscience*.

[51] van den Biggelaar AHJ, Gussekloo J, de Craen AJ (2007). Inflammation and interleukin-1 signaling network contribute to depressive symptoms but not cognitive decline in old age. *Experimental Gerontology*.

[52] Wieseler-Frank J, Maier SF, Watkins LR (2005). Immune-to-brain communication dynamically modulates pain: physiological and pathological consequences. *Brain, Behavior, and Immunity*.

[53] McNally L, Bhagwagar Z, Hannestad J (2008). Inflammation, glutamate, and glia in depression: a literature review. *CNS Spectrums*.

[54] Saito K, Crowley JS, Markey SP, Heyes MP (1993). A mechanism for increased quinolinic acid formation following acute systemic immune stimulation. *The Journal of Biological Chemistry*.

[55] Wöhrl R, Eisenach S, Manahan-Vaughan D, Heinemann U, von Haebler D (2007). Acute and long-term effects of MK-801 on direct cortical input evoked homosynaptic and heterosynaptic plasticity in the CA1 region of the female rat. *European Journal of Neuroscience*.

[56] Rinaldi T, Perrodin C, Markram H (2008). Hyper-connectivity and hyperplasticity in the medial prefrontal cortex in the valproic acid animal model of autism. *Frontiers in Neural Circuits*.

